# Polyoxometalate Nanoparticles as a Potential Glioblastoma Therapeutic via Lipid-Mediated Cell Death

**DOI:** 10.3390/ijms23158263

**Published:** 2022-07-27

**Authors:** Michael S. Petronek, Bryan G. Allen, Gregor Luthe, Jeffrey M. Stolwijk

**Affiliations:** 1 Department of Radiation Oncology, Division of Free Radical and Radiation Biology, The University of Iowa, Iowa City, IA 52242-1181, USA; 2 Spheres4Life B.V., 7521 Enschede, The Netherlands; bryan-allen@uiowa.edu (B.G.A.); gregor.luthe@nb-gmbh.com (G.L.)

**Keywords:** nanoparticles, polyoxometalate, glioblastoma, cell culture

## Abstract

Polyoxometalate nanoparticles (POMs) are a class of compounds made up of multiple transition metals linked together using oxygen atoms. POMs commonly include group 6 transition metals, with two of the most common forms using molybdenum and tungsten. POMs are suggested to exhibit antimicrobial effects. In this study, we developed two POM preparations to study anti-cancer activity. We found that Mo-POM (NH_4_)Mo_7_O_24_) and W-POM (H_3_PW_12_O_40_) have anti-cancer effects on glioblastoma cells. Both POMs induced morphological changes marked by membrane swelling and the presence of multinucleated cells that may indicate apoptosis induction along with impaired cell division. We also observed significant increases in lipid oxidation events, suggesting that POMs are redox-active and can catalyze detrimental oxidation events in glioblastoma cells. Here, we present preliminary indications that molybdenum polyoxometalate nanoparticles may act like ferrous iron to catalyze the oxidation of phospholipids. These preliminary results suggest that Mo-POMs (NH_4_)Mo_7_O_24_) and W-POMs (H_3_PW_12_O_40_) may warrant further investigation into their utility as adjunct cancer therapies.

## 1. Introduction

Polyoxometalate nanoparticles (POMs) are compounds that consist of multiple (three or more) transition metal oxyanions that are linked together using oxygen atoms. POMs most commonly include group 6 transition metals, but may also contain group 5 metals [1,2]. Two of the most commonly utilized POMs contain the group 6 transition metals tungsten (W) and molybdenum (Mo). The transition metals contained within a POM are typically in their highest oxidation state. POMs are frequently utilized as chemical catalysts; both Mo and W are utilized in proteins for electronic processes [1,2]. An example of this is molybdopterins, which are present in enzymes such as xanthine oxidase, a superoxide-generating enzyme that catalyzes the oxidation of hypoxanthine to xanthine for the formation of uric acid [3,4]. Similarly, carboxylic acid reductase contains a W-pterin complex that catalyzes the reduction of carboxylic acids to aldehydes [5]. Thus, the apparent redox activity in biological systems of both W and Mo may be exploited therapeutically.

From a therapeutic perspective, POMs may have antimicrobial properties that can be used to treat mycoplasma infections [6,7]. We hypothesized that high-dose POMs may also be a potent cancer therapeutic due to the biological redox activity of W and Mo [7,8]. Mo-POMs have recently been found to induce apoptosis in cancer cells [9,10]. Sodium molybdates can also alter iron metabolism and induce cell death in cancer cells [10]. However, the characterization of their effects on biological processes in cancer cells remains limited. In this study, we assessed the effects of ammonium molybdate (NH_4_)Mo_7_O_24_) and phosphotungstate (H_3_PW_12_O_40_) POMs (Mo- and W-POMs, respectively) on glioblastoma cells and provide preliminary evidence of their effects on lipid peroxidation, which has yet to be tested and can open a window of opportunity for deeper mechanistic studies and therapeutic strategies. POMs are frequently used as hybrid nanoparticles or as scaffolds for POM-drug composites using traditional cancer therapies (e.g., 5-fluouridine) [11]; however, we are investigating independent POM nanoparticles in order to begin to gain a deeper understanding of their contributions to cancer therapy. Because catalytically active metals (e.g., iron) can readily catalyze lipid peroxidation, we focused our attention on the ability of Mo- and W-POMs to promote lipid peroxidation. 

Membrane lipid peroxidation (LPO) can be initiated when a strong 1-electron oxidant reacts with a polyunsaturated phospholipid, Equation (1). This event results in the formation of a carbon-centered phospholipid radical (PL^●^) that rapidly reacts with oxygen to yield a phospholipid peroxyl radical (PLOO^•^), Equation (2), [12]. This radical can generate new chains of lipid oxidation reactions. The abstraction of a hydrogen atom by PLOO^•^) results in the formation of a phospholipid hydroperoxide (PLOOH), Equation (3).

PL-H + Oxidant^●^ ➔ PL^●^ + Oxidant-H (1)

PL^•^ + O_2_ ➔ PLOO (2)

PLOO^•^ + PL-H ➔ PL^•^ + PLOOH (3)

When in the presence of ferrous iron, phospholipid hydroperoxides form a phospholipid alkoxyl radical Equation (4).

PLOOH + Fe^2+^ ➔ PLO^•^ + OH^−^ + Fe^3+^ (4)

The reactive alkoxyl radical, PLO^•^, can lead to new chains of lipid peroxidation in membranes, typically via epoxyperoxyl radicals [13,14,15]. However, the extent by which other metals can catalyze the formation of alkoxyl radicals is poorly understood and currently understudied. Therefore, we hypothesized that Mo- and W-POMs may be able to catalyze the formation of alkoxyl radicals to promote cellular lipid peroxidation. 

## 2. Results

### 2.1. UV-Vis Determination and Stability of POMs

For our studies, we first investigated how to properly prepare and store POM stocks for use in cell culture. Since Mo-POM and W-POMs are acidic, we have made three different solutions:

1. 100 mM POMs in NanoPure water;
2. 100 mM POMs in 50 mM KPO4 buffer, pH attempted to adjust;
3. 100 mM POMs in 0.5 M HEPES buffer, pH adjusted to pH ≈ 6–7 (Table 1).

The POMs dissolved readily in water. However, it was too acidic to achieve final concentrations > 100 µM in cell culture medium. Therefore, we dissolved the POMs in HEPES buffer. The concentration of HEPES in the stock would be an insignificant in the cell culture medium. The pH was adjusted with 5 N NaOH and checked with pH-paper to be approximately 7. 

**Figure 1:**
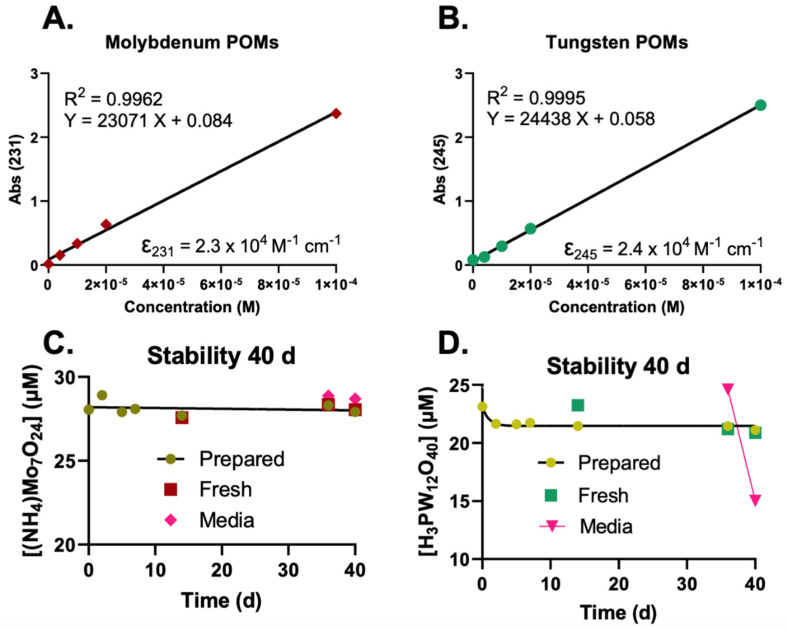
Molybdenum and tungsten containing polyoxometalates are stable in aqueous and buffered solutions. (**A**,**B**) Molybdenum ((NH_4_)Mo_7_O_24_) and tungsten (H_3_PW_12_O_40_) polyoxometalates were prepared (2–100 µM). The relative absorbances were plotted in an XY-plot where the slope represents the molar attenuation coefficient. (**C**,**D**) A 100 mM (in 0.5 M HEPES) stock solution for each POM on day 1 to test for stability. The stock was diluted to 1 mM in water to also obtain information about the stability of the POMs in aqueous environments. The aqueous solutions were further diluted to approximately 25 mM to be in an appropriate absorbance range (<1 Abs) on day 1 (Prepared). The stability was tracked for 14 days, after which a new dilution was made from the 100 mM stock (Fresh). The Mo-POM (**C**) concentration appears stable in both the 100 mM (in 0.5 M HEPES) solution in a lighted lab environment, as well as in an aqueous solution. After 35 days, a 1 mM solution was made in complete medium, then diluted to 25 mM. The solution was kept in an incubator for 4 days to reflect a maximum treatment time. The Mo-POM was both measurable in medium and stable for at least 4 days. The W-POM (**D**) concentration of the aqueous dilution dropped from ≈23 µM to ≈21 µM in the first few days. Making a fresh dilution (d = 14) shows the same effect. In contrast to the molybdenum POM, the W-POMs appear to be less stable in media over a time of 4 days, whereas the observed concentration dropped from ≈25 µM to ≈15 µM.

To determine the stability of the POMs, UV-Vis spectra sweeps were taken between 200 and 600 nm using the Implen^TM^ P 330 NanoPhotometer (Figure 1A,B). The relative absorbances were plotted in an XY-plot and the linearity was confirmed with R^2^ values > 0.99. The plotted linear regression slope represents the molar attenuation coefficient, which is further used to determine the concentration of POMs for the stability experiments. 

Three different solutions of POMs were tested for stability.

- The high concentration HEPES stock (100 mM POM in 0.5 M HEPES);
- 1 mM stock in water (100× dilution of the HEPES stock in water);
- 1 mM stock in medium (100× dilution of the HEPES stock in medium).

The 100 mM POM-HEPES was prepared on day 1 (Figure 1C,D) and stored at room temperature in a glass 15 mL vial. To obtain information about the stability of the POMs in aqueous environments, the 1 mM stock in water was used. This aqueous solution was diluted to approximately 25 mM to be in an appropriate absorbance range (<1 Abs) on day 1 following preparation. The stability was tracked for 14 days, after which a new dilution was made from the 100 mM POM-HEPES stock (Fresh). The Mo-POM concentration appears stable in the 100 mM stock and an aqueous solution at a lower concentration (1 mM). After 35 days, a 1 mM solution was made in complete medium (DMEM F-12 media containing 15% FBS, 1% penicillin-strep, 1% Na-pyruvate, 1.5% HEPES, 0.1% insulin, and 0.02% fibroblast growth factor), where the POMs were diluted to 25 mM. The solution was kept in a 37 °C incubator for four days to reflect an extended treatment time of POMs for a typical cell culture experiment. The Mo-POM was stable for at least four days. The aqueous dilution’s W-POM (D) concentration dropped from ≈23 µM to ≈21 µM in the first four days. This did not occur in the 100 mM POM-HEPES stock, as the “Fresh” sample at day 14 appears to still be ≈23 µM. In contrast to the Mo-POM, W-POMs appear to be less stable in media over four days with a concentration decrease of ≈25 µM to ≈15 µM. 

### 2.2. Cytostatic and Cytotoxic Effects of POMs in Glioblastoma Cells

**Figure 2:**
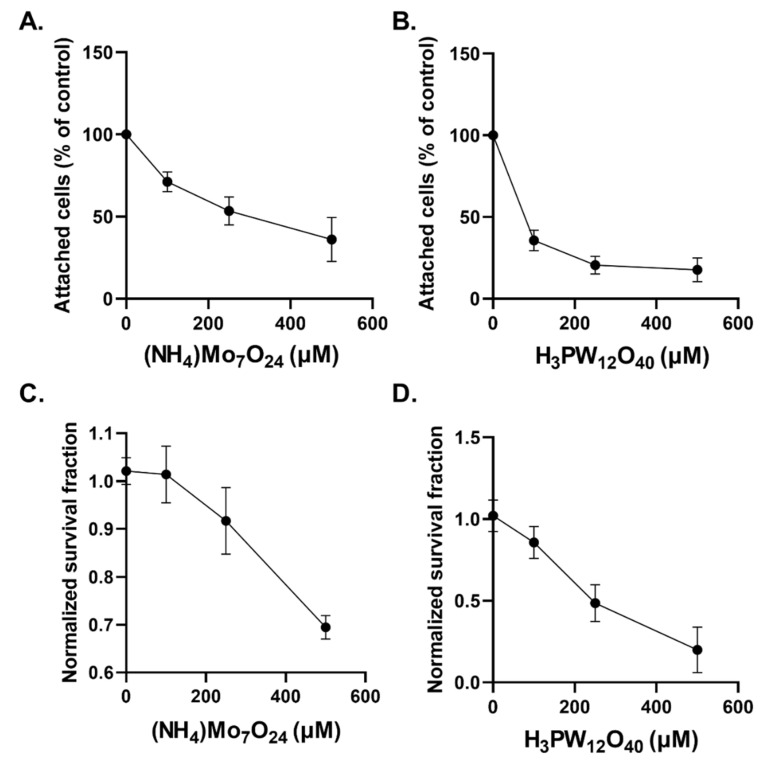
Tungsten- and molybdenum-containing POMs have cytostatic and cytotoxic effects in GBM cells. The number of attached cells were counted following a 48 h treatment of U87 glioblastoma cells treated with increasing concentrations of (NH_4_)Mo_7_O_24_ (**A**) and H_3_PW_12_O_40_ (**B**). Following the 48 h treatment with increasing concentrations of (NH_4_)Mo_7_O_24_ (**C**) and H_3_PW_12_O_40_ (**D**), U87 cells were replated as single cells to evaluate their clonogenic potential.

The investigation of POMs in cancer cell biology has recently garnered interest but remains limited. We first aimed to evaluate both the cytostatic and cytotoxic effects of Mo-POMs ((NH_4_)Mo_7_O_24_) and W-POMs (H_3_PW_12_O_40_). We observed that increasing concentrations of Mo-POMs for an extended 48 h period resulted in a concentration-dependent growth delay (Figure 2A). A similar concentration-dependent growth delay was observed with W-POMs (Figure 2B). W-POMs appear to have more dramatic effects on glioblastoma cells than Mo-POMs as 100 µM was able to stall growth by over 50%, but the same level of growth inhibition was not achieved until 500 µM concentrations of NH_4_Mo_7_O_24_ were reached. Similarly, we observed a Mo- and W-POM concentration-dependent decrease in clonogenic survival (Figure 2C,D). Similar to the growth delay results, the cytotoxic effects of W-POMs greatly exceeded the effects of Mo-POMs as they failed to reach 50% cell killing whereas the effect was achieved with 250 µM W-POMs. These data show that high-dose Mo-and W-POMs (≥100 µM) exhibit cytostatic and cytotoxic effects on glioblastoma cells. 

### 2.3. Morphological Changes Associated with POMs Treatment

**Figure 3:**
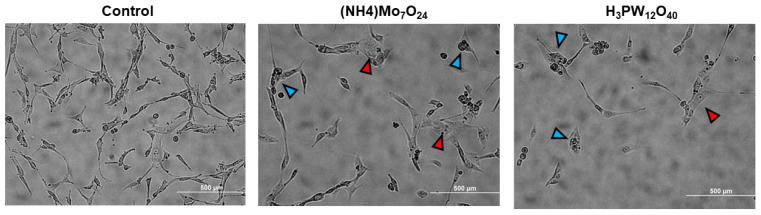
POM-induced morphological changes. Following 48 h treatment of U87 cells with 500 µM (NH4)Mo_7_O_24_ and H_3_PW_12_O_40_ light microscopic images were collected to visualize morphological changes associated with the treatment. Blue arrows indicate multi-nucleated cells or cells with cellular fragmentation and red arrows indicate cellular swelling.

Following 48 h treatment, we characterized the morphological changes of U87 cells using light microscopy (Figure 3). Evaluation of cells with a light microscope revealed significant morphological changes compared to the controls, initially marked by a decrease in cell density, consistent with the cytostatic effects of Mo- and W-POMs. We also observed significant cell membrane swelling compared to the control cells and the presence of multi-nucleated cells and/or cellular fragmentation. These two critical morphological changes may indicate the activation of necrosis (cellular swelling) along with mitotic catastrophe or apoptotic events [16,17,18]. These findings would provide two potential cell death mechanisms induced by Mo- and W-POMs in glioblastoma cells. 

### 2.4. Mo-POMs Promote Lipid Peroxidation in GBM Cells

**Figure 4:**
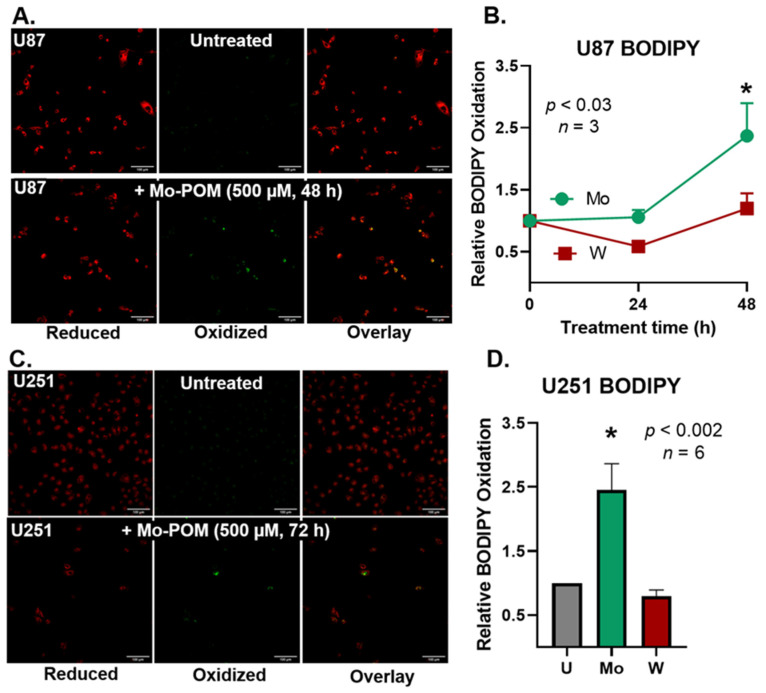
Molybdenum-POMs promote lipid peroxidation in U87 and U251 GBM cells. (**A**) U87 GBM cells cultured in BF15 DMEM medium and treated with 500 µM of Mo-POM (48 h). Incubated for 30 min with BODIPY C11 after treatment. The probe was handled in the dark. There are low amounts of oxidation (green) in the untreated cells, whereas the Mo-POM-treated cells show increased green signals. (**B**) U87 GBM cells treated for 24 or 48 h with Mo- and W-POMs at 500 µM. Three biological replicates were analyzed and plotted. A period of 48 h treatment of Mo-POM increases BODIPY oxidation, reflecting lipid oxidation. However, W-POM does not appear to have any effect, and at 24 h is trending to be more reducing. (**C**) U251 GBM cells cultured in BF15 DMEM medium and treated with 500 µM of Mo-POM (72 h). Incubated for 30 min with BODIPY C11 after treatment. The probe was handled in the dark. There are low amounts of oxidation (green) in the untreated cells, whereas the Mo-POM-treated cells show increased green signals. (**D**) U87 GBM cells treated for 72 h with Mo- and W-POMs at 500 µM. Six biological replicates were analyzed and plotted. 72 h treatment of Mo-POM increases BODIPY oxidation, reflecting lipid oxidation. However, W-POM does not appear to have any effect and is trending to be more reducing. * *p* < 0.05 using a one-way ANOVA test.

Because Mo- and W-POMS are redox-active and catalytically-active metals (e.g., iron) are well known to induce lipid peroxidation [19,20,21,22,23,24], we hypothesized that Mo- and W-POMs may also be inducing detrimental lipid oxidation events. U87 cells were plated and measured following an extended 24 and 48 h treatment with 500 µM (NH_4_)Mo_7_O_24_ and H_3_PW_12_O_40_ (Figure 4A,B). We observed a significant increase in lipid oxidation associated with Mo-POMs after 48 h of treatment. In contrast, H_3_PW_12_O_40_ treatment did not increase BODIPY oxidation at 24 or 48 h. These data suggest that detrimental lipid oxidations may also play a critical role in the cytotoxic effects of Mo-POMs in glioblastoma cells and may induce a ferroptosis–like cell death mechanism. 

To probe the generality of this effect, we used a second GBM cell line, U251. The cells were plated and treated with both POMs for 72 h because we observed the temporal increase in BODIPY oxidation in U87 cells and aimed to exacerbate the effect. After 72 h of treatment, a similar trend was found when compared to the U87 cells (Figure 4C,D). We observed Mo-POMs induce the oxidation of BODIPY, whereas W-POMs do not. These data suggest that Mo-POMs may kill cells via a lipid peroxidation pathway whereas W-POMs do not, despite their superior toxicity. Therefore, Mo- and W-POMS may be utilizing independent cell-killing mechanisms. 

## 3. Discussion

In this work, we created an easy method to generate a stock solution of POMs for cell culture use. In addition, we identified a simple way to quantify POMs in solution using UV-Vis spectroscopy. However, the maximum linear absorbances of both Mo and W- POMs are in relatively low UV wavelengths (231 and 245 nm). Therefore, NanoPhotometers or quartz cuvettes should be used to translate this technique to other laboratories. The largest benefit of this method is a relatively high extinction coefficient (2.3 × 10^4^ and 2.4 × 10^4^ mol^−1^ cm^−1^), resulting in very low sensitivity. Short-term shelf life of the 100 mM POM stocks appears to be sufficient for multiple experiments. However, the long-term stability of the 100 mM stocks should be further investigated. Furthermore, the W-POM nanoparticles in cell culture media appear to be unstable and may dissociate. At first glance, W-POMs are very toxic to GBM cells. However, it is unclear whether tungsten oxides remain in the medium or act as dissolved metal oxides and further analysis is required. Optimizing concentrations of POMs in cell culture media is necessary to individualize therapy (i.e., low dose: antibiotic agent; high dose: anti-cancer agent). Purely inorganic POMs, such as those used in this study, have been reported to have limited cell penetration and stability in solution, which has led to the increased interest in use of POMs in organo-metallic compounds for cancer therapy. Several different classes of organo-metallic compounds are being investigated currently for use as cancer therapeutics due to their increased stability in solution and cell penetration along with reduced toxicity; these are extensively reviewed in [11].

We observed both cytostatic and cytotoxic effects in U87 glioblastoma cells associated with POM treatment. While significant mechanistic work is required to evaluate the anti-cancer effects of both Mo- and W-POMs, we observed significant morphological changes that may be indicative of apoptosis activation and mitotic cell death. Recently, it has been shown that organically-derivatized Mo-based POMs can cause similar morphological changes in U251 glioblastoma cells, which were also associated with purported apoptosis induction [9]. We also observed an increase in lipid oxidation events associated with (NH_4_)Mo_7_O_24_ POM treatment. This may result from extracellular reactive oxygen species generation as Mo-POMS can undergo redox reactions [19,24,25]. In this manner, Mo-POMS may be mimicking iron in its ability to catalyze the formation of alkoxyl radicals (Figure 4) in what is now commonly referred to as ferroptosis [14,20,22]. Interestingly, W-POM appears to be more toxic than Mo-POM. These data suggest that the mechanism of cell death is not primarily due to lipid peroxidation. This finding is of particular importance because U251 cells are considerably more resistant to ionizing radiation than other glioblastoma cell lines, and POM-based nanoparticle platforms may provide a novel therapeutic outlet to overcome resistance [26]. Thus, both Mo- and W-POMs may have therapeutic potential in glioblastoma cells by serving as a reserve of redox-active metals to induce cell death. However, the POM-independent effects in the initiation of lipid peroxidation suggest that each POM provides different mechanistic properties to their anti-cancer effects. Therefore, it may be pertinent to consider each POM carefully when designing POM-drug composites for anti-cancer therapy. Purely inorganic POMs have been reported to enhance cellular reactive oxygen species to have anti-cancer effects [27]; however, clinical and pre-clinical toxicities have been reported that must be taken into consideration when evaluating their utility in cancer therapy [28,29]. Malignant brain tumors have been reported to have altered antioxidant capacity and increased oxidative stress compared to normal brain tissue. Thus, POMs may exhibit intrinsic tumor selectivity by functioning as a redox-based therapeutic strategy, but special attention must be paid to their potential toxicity profile [30]. Simultaneously, lower doses of POMs did not have an effect on GBM cells (Figure 2), suggesting a therapeutic window to explore POM anti-microbrial properties. Additional research needs to be conducted on the potential mechanism(s) and tumor-specificity of cell killing associated with each POM. 

## 4. Materials and Methods

### 4.1. POM Preparation

A relative high stock concentration (10–100 mM) is needed to limit the total volume of the stock added to the media. Currently, there is limited information available regarding the toxicity of POMs on cells and tissues and the underlying mechanisms driving these effects. Therefore, we have decided to make the following stocks to investigate these. 

#### 4.1.1. Reagents

For the assay, the following stock solutions or reagents are to be available:

- Gibco HEPES buffer 1 M (catalog#: 15630080).
- Tungsten POM (phosphotungstic acid hydrate, Sigma-Aldrich); molecular weight = 2880.05 g mol^−1^.
- Molybdenum POM (ammonium heptamolybdate tetrahydrate, Sigma-Aldrich) molecular weight = 1235.86 g mol^−1^.
- NaOH 5 N solution.

#### 4.1.2. Materials

- Glass HPLC vials (1.5 mL).
- Small glass Erlenmeyer flask/beaker or volumetric flask (10–25 mL).
- pH paper or pH meter.

The final concentrations of the POM-mixtures will be as followed (Table 1):

1. Start by calculating the amount of POM powder needed. Use the basic molar calculation equations.
2. Weigh out the amount calculated above.
3. Dissolve the POMs in half the total volume of 1 M HEPES buffer, e.g., if you want to make 10 mL POM stock, dissolve the POMs in 5 mL of 1 M HEPES buffer.
4. While stirring, gently add the 5 N NaOH. For 10 mL stock, 1 mL of NaOH is needed for Mo-POMs, and 1.2 mL for W-POMs.
5. Add ddH_2_O totaling to your desired volume.
6. Test the pH of the stock using pH paper or a pH meter. The pH should be around 6–7.
7. Store aliquots and the main stock solution only in glass since the POMs may stick to plastic in high concentration solutions.

### 4.2. UV-Vis Spectroscopy

All spectrophotometric measurements were carried out using a microvolume spectrophotometer (Implen^TM^ P 330 NanoPhotometer^®^; Munich, Germany) instead of a normal cuvette spectrophotometer. The Implen NanoPhotometer works by decreasing the pathlength, and thus introducing an artificial dilution factor so less sample is needed. More importantly, there is a direct measurement of the POM samples, eliminating any interference of any cuvette material. 

### 4.3. Cell Culture

U87 and U251 glioblastoma cells were cultured at 21% O_2_ in DMEM F-12 media containing 15% FBS, 1% penicillin-strep, 1% Na-pyruvate, 1.5% HEPES, 0.1% insulin, and 0.02% fibroblast growth factor. Cells were plated in 60 mm^2^ dishes and grown to 70–80% confluence prior to experimentation. Cells were treated with POMs in complete cell culture media. 

### 4.4. BODIPY C11 Labeling of Cells 

To test the effects of POMs on lipid oxidation events, we performed BODIPY C11 confocal imaging on U87 and U251 glioblastoma cells. BODIPY C11^581/591^ is a lipophilic redox probe that changes fluorescence emission upon oxidation. The probe is designed with a long carbohydrate chain (C11) to facilitate its distribution into the lipid regions of cellular membranes [31]. Oxidation of the probe is correlative to the flux of PLOO^•^, which is a function of the PUFA content of membranes, i.e., PUFA content modulates the methylene bridge index (MBI) [32] and are very oxidizable. 

**Figure 5:**
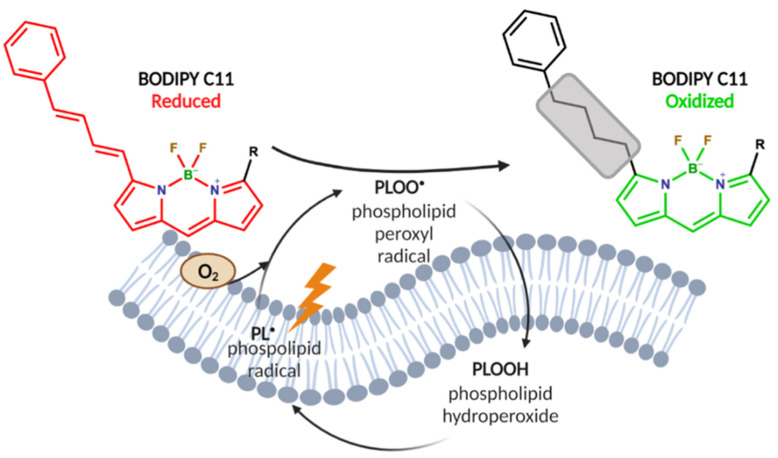
Mechanism of BODIPY oxidation by lipid radicals. LPO occurs when lipid radicals react with oxygen, cycling to phospholipid peroxyl radical and phospholipid hydroperoxide. This probe was designed to migrate to lipid cellular membranes, where R = 11 carbon chain, hence C11. In its reduced state, BODIPY emits fluorescence red light due to its conjugated diene phenyl structure. Upon oxidation, by phospholipid peroxyl radical, the diene chain loses conjugation, therefore preventing localization of electrons in this region. This shifts the fluorescent emission from red to green.

In its reduced form, BODIPY will emit in bright red (λ ≈ 590 nm) upon excitation. It is suggested that the diene moieties are the main oxidation sites. This removes the conjugation from the diene phenyl moiety of BODIPY. Several oxidation products have been proposed but remain largely unidentified. The loss of conjugation limits the electron delocalization to the boron dipyrromethene difluoride core, shifting the fluorescence emission to a shorter wavelength, (green, λ ≈ 520 nm). Phospholipid peroxyl radicals (PLOO^•^) will bring about the oxidation of BODIPY, Figure 5. Thus, BODIPY oxidation reports on the flux of PLOO^•^ in the system. 

BODIPY C11 (ThermoFisher D3861) (5 mM) in DMSO was prepared in the dark and stored at −20 °C. Cells were cultured in 60 mm dishes until 70–80% confluency was reached. Then, the cells were washed twice with PBS or HBSS. Avoiding any source of light, BODIPY C11 was diluted to 1 µM in phenol-free medium, containing 10% fetal bovine serum. Finally, the plates were incubated at 37 °C for 30 min prior to measurement. This incubation time facilitates the migration of BODIPY C11 into the cellular membranes. After the incubation, the samples were analyzed using confocal laser scanning microscopy.

Confocal images of living cells were obtained using an Olympus FV1000 laser scanning confocal microscope. BODIPY C11 emissions were captured at 515 nm (oxidized) and 543 nm (reduced) in sequence with a 20× water immersion objective immersed directly into phenol red-free media. Images were analyzed using ImageJ by taking the ratio of the mean fluorescence of both channels (green and red). If the lipids in cells are more oxidized, the green channel will be brighter, thus increasing the ratio. 

### 4.5. Statistics 

Results are expressed in mean ± SD (unless specified otherwise), where *p* < 0.05 was considered significantly different. Statistical analyses were performed using a One-way analysis of variance to test for differences in means, or where appropriate the unpaired student’s *t*-test. Linear regression was used to determine slopes. Calculations were performed using Prism for Mac OS X, Version 9.3 (GraphPad Software, San Diego, CA, USA). 

## 5. Conclusions

In summary, we have been able to develop an easy-to-use, UV-Vis spectroscopy-based method to accurately observe (NH_4_)Mo_7_O_24_ and H_3_PW_12_O_40_ POMs that can be utilized in culture media. The Mo-POM is stable in stock solutions (100 mM POM in 0.5 M HEPES), as well as in water diluted (25–1000 µM POMs) samples for at least 40 days when stored in typical laboratory environments. Additionally, Mo-POMs (1 mM) are stable in cell culture media for at least 96 h of incubation time at 37 °C. However, W-POM did show a small reduction in concentration a few days after dilution into water (1 mM). In addition, the observed concentration of W-POM in cell culture media was decreased by approximately 40% after 4 days. The mechanism of this reduction must be further investigated. 

We have also provided preliminary observations regarding the cytostatic and cytotoxic effects of these POMs in glioblastoma cells. Interestingly, we have shown POM-specific effects with enhanced intrinsic glioblastoma to W-POMs relative to Mo-POMs. Despite the increased toxicity, W-POMs in GBM did not exhibit any lipid oxidation and in some cases even appeared more reducing than its untreated equivalent. However, lipid peroxidation was observed in two GBM cell lines, U87 (48 h) and U251 (72 h) treated with 500 µM Mo-POM. This provided preliminary mechanistic evidence linking (NH_4_)Mo_7_O_24_ POMs to the catalysis of lipid oxidation events. Thus, while future mechanistic studies are required, Mo- and W-POMs may have therapeutic potential in glioblastoma cells using POM-specific, non-generalizable mechanisms. Alongside this research, other therapeutic avenues can be explored in the future.

## Figures and Tables

**Table 1:** Final concentrations of the POM stock solutions.

Element	[HEPES] (mM)	[POM] (mM)	[NaOH] (mM)
Tungsten	500	100	600
Molybdenum	500	100	500
